# Association of IL-22 and IL-22RA1 gene variants in Iranian patients with colorectal cancer 

**Published:** 2021

**Authors:** Seyed Reza Mohebbi, Khatoon Karimi, Fatemeh Rostami, Shabnam Kazemian, Pedram Azimzadeh, Hanieh Mirtalebi, Ehsan Nazemalhosseini-Mojarad, Hamid Asadzadeh Aghdaei, Reza Vafaee, Mohammad Hossain Heydari

**Affiliations:** 1 *Gastroenterology and Liver Diseases Research Center, Research Institute for Gastroenterology and Liver Diseases, Shahid Beheshti University of Medical Sciences, Tehran, Iran *; 2 * Basic and Molecular Epidemiology of Gastrointestinal Disorders Research Center, Research Institute for Gastroenterology and Liver Diseases, Shahid Beheshti University of Medical Sciences, Tehran, Iran *; 3 * Foodborne and Waterborne Diseases Research Center, Research Institute for Gastroenterology and Liver Diseases, Shahid Beheshti University of Medical Sciences, Tehran, Iran*; 4 *Laser Application in Medical Sciences Research Center, Shahid Beheshti University of Medical Sciences, Tehran, Iran*; 5 * Proteomics Research Center, Faculty of Paramedical Sciences, Shahid Beheshti University of Medical Sciences, Tehran, Iran*

**Keywords:** Colorectal cancer, Interleukin-22 gene, Interleukin-22 receptor A1 gene, Single nucleotide polymorphism, PCR-RFLP

## Abstract

**Aim:**

In the current study, it was hypothesized that single nucleotide polymorphisms (SNPs) in the regulatory region of the IL-22 signaling pathway genes, including IL-22 and IL-22RA1 variants, may be associated with CRC susceptibility.

**Background:**

The important role of pro-inflammatory cytokines during tumorigenesis is well-established. In recent years, IL-22 has been linked with colorectal cancer (CRC) through a number of mechanistic and observational studies

**Methods:**

The association of four polymorphisms in the IL-22 (rs1179251 and rs1179246) and IL-22RA1 (rs4648936 and rs10794665) genes with CRC risk were studied using a case-control design with 304 cases and 345 controls from the Iranian population. All 649 subjects were evaluated by PCR–RFLP method.

**Results:**

No significant difference was found in genotype and allele frequencies between the cases and controls for either IL-22 and IL-22RA1 gene variants or CRC risk before or after adjusting for confounders.

**Conclusion:**

The current findings do not present any significant evidence for associations between variants in IL-22 signaling pathway genes and CRC. Complementary studies with greater sample sizes may be necessary to fully elucidate the nature of these associations.

## Introduction

 Colorectal cancer (CRC) is known as the third most common malignancy and fourth most frequent cause of cancer mortality worldwide (1). It is also one of the leading causes of cancer deaths in developed countries (2, 3). Various factors have been determined to be responsible for individual susceptibility to CRC including inheritance, environmental factors (e.g., composition of diet, obesity, diabetes mellitus, smoking, alcohol consumption), and chronic intestinal inflammation (4). Since chronic inflammation influences tumor development through its ability to induce mutations (e.g., reactive oxygen or nitrogen species) (5), recent data has proposed a direct effect of inflammation on tumor growth. IL-22 is characterized as a member of the IL-10 family. It is a class II α-helical proinflammatory cytokine expressed by stimulated T cells and activated natural killer cells (6). The receptor for IL-22 is comprised of the IL-22R1 chain and IL-10 receptor 2 (IL-10R2) heterodimeric complex (7). Despite its structural similarity to IL-10 (between 20% and 28%), immune cells are not the target cells of IL-22. IL-22Rα expression is limited to specific tissues such as the colon, liver, kidney, skin, pancreas, and lungs (8-13). It has been shown that IL-22R1 is broadly expressed in the colon, pancreas, and skin in addition to many tumor cell lines derived from these tissues (7). Therefore, it is likely that signaling activated by IL-22 leads to pro-tumor effects. Previous surveys have demonstrated the influence of IL-22 on the development of CRC (14). It seems that IL-22 has a profound influence on colonic epithelial cell proliferation (15). In addition, IL-22 can effectively promote IL-10 secretion from intestinal epithelial cells, and it may lead to immunomodulation conditions in CRC (16). Furthermore, IL-22 can induce several genes that are associated with the maintenance of CRC cancer stem cells by promoting the STAT3 related pathway and epigenetic activation of these genes (17, 18).

Additionally, only one study that has evaluated the association between IL-22 polymorphism and risk of CRC (19). To identify genetic factors for CRC, two variants within the intron 4 and the 3’ untranslated region (UTR) of the IL-22 gene, rs1179251 and rs1179246 (located at chromosome 12) and two variants in the intron 6 and 3’near gene of the IL-22Rα gene, rs4648936 and rs10794665 (located at chromosome 1) with the risk of CRC were evaluated. Because of its location in the regulatory region of the IL-22 and IL-22Rα genes, it was postulated that these SNPs have the potential to influence IL-22 expression or serve as a surrogate for unrecognized neighboring functional variations. These analyses, therefore, serve as an initial evaluation of the potential importance of IL-22 and IL-22RA1 gene polymorphisms in CRC susceptibility. 

**Table 1:** Detection of single nucleotide polymorphisms (SNPs) within the IL-22 and IL-22Rα genes

Gene	dbSNP^1^	Region^2^	PCR primers (5’ 3’)	Product size (bp)	Restriction enzyme	Digestion temp.(h)^3^	Allele size (bp)^4^
IL-22	rs1179251	intron 4	F: CCAGAAATTAGCCCTATATGC	560	AlwNI	37°C (3)	AlleleG:560
			R: GAAGGACACAGTGAAAAAGGTAGG				AlleleC:299,261
	rs1179246	3’UTR	F: AAGGGAAGACTCACTGTTCTGA	426	AluI	37°C (3)	AlleleA:325,101
			R: CTGGTGGGCTGAAAGGTGT				AlleleC:239,101,89
IL-22RA1	rs4648936	intron 6	F: GAGACATGTGTTGTGTGGAGCC	305	BsmAI	55°C (3)	AlleleG:305
			R: TCCCTTTAGACCCTCAGCC				AlleleA:193,112
	rs10794665	3’near gene	F:GAGAAGGTTCCACATCATTTGTC	242	AluI	37°C (3)	AlleleG:223, 19
			R: AGTGCCCAATAAACGGTAGC				AlleleA:137,86,19

^1^ Positions: rs1179251: chr12:68251271 (GRCh38.p13), rs1179246: chr12:68246803 (GRCh38.p13), rs4648936: chr1:24141971 (GRCh38.p13), rs10794665: chr1:24119638. ^2 ^Location of the polymorphism. ^3^ Temperature in °C and duration of digestion time (hours). ^4^ Length of digestion products for each allele in base pair.

## Methods

**Study subjects**

A total of 649 individuals who were subjected to colonoscopy for different gastrointestinal problems or checkup were called up by the Research Institute for Gastroenterology and Liver Diseases of the Shahid Beheshti University of Medical Sciences and checked in a cross-sectional, case-control study. The colorectal cancer patient group comprised 304 subjects (age range 24-92 years) and was defined as patients with positive pathological reports for CRC; control participants comprised 345 subjects (age range 13-86 years) who showed no signs of malignancy or polyps (adenomatous or other) in their colonoscopy results. Informed consent was provided by all subjects at recruitment, and the Ethical Review Board of the research institute approved the study protocol.

**DNA analysis**

Total DNA was extracted and isolated from whole-blood using a standard protocol for extraction with phenol-chloroform. For each studied gene, the variation site was amplified by a polymerase chain reaction (PCR) with a final volume of 25 μl. Then the PCR product was digested by a corresponding restriction enzyme using the restriction fragment length polymorphism technique. Primer sets, annealing temperatures, and restriction enzymes (AluI and BamAI; Thermo Scientific, Lithuania, AlwNI; NEB, MA, USA) used for the PCR-RFLP assay are shown in Table 1. Each product and allele digested fragment size was determined. PCR cycling was performed as follows: initial denaturation step at 94 ℃ for 5 min, followed by 35 cycles of denaturation at 94 ℃ for 45 s, annealing for 40 s and extension at 72 °C for 45 s, with a final extension of 5 min at 72 °C. Then, 15 μl of the PCR product was then cleaved with a corresponding restriction enzyme. The PCR and restriction enzyme products were analyzed by electrophoresis in a two- and three-percent agarose gel, respectively, and subsequently stained with ethidium bromide to be visualized under exposure of UV light. Additionally, to confirm the PCR-RFLP assay, about 5% of total samples for each locus were randomly selected, and then the PCR products of each were sequenced on ABI genetic analyzer 3130xl. 

**Statistical methods**

The distribution of genotypes in cases and controls was tested for a departure from Hardy-Weinberg equilibrium by means of the χ^2^ test, which was also used to examine the relative association between patients and controls for genotype and allele frequencies. To adjust confounding factors including age, BMI, sex, and smoking status, logistic regression analysis was used. Odds ratios (OR) and 95% confidence intervals (95% CI) were used to estimate the association between individual polymorphisms and colon cancer. The t-test was used to evaluate variations in demographic factors. A *p*-value less than 0.05 was considered to be statistically significant. All statistical analyses were performed using SPSS (version 15.0; SPSS, Chicago, IL, USA). 

**Table 2:** General characteristics of the studied population

Variables	Controls (n=345)	Cases (n=304)	Pvalue
Age (Mean ± SD)	45.2 ± 17.1	57.7 ± 12.2	<0.0001
BMI^*^ (kg/m^2^)	26.1 ± 5.5	25.6 ± 4.9	0.112
Gender, n (%) Female Male			
	166 (48.12%)	140 (46.05%)	0.451
	179 (51.88%)	164 (53.95%)	
Smoking Smoker Non-Smoker			
	23 (6.70%)	37 (12.17%)	0.110
	322 (93.30%)	267 (88.83%)	
Tumor Location Colon Rectum			
	_	218 (71.71%) 86 (28.29%)	-

* BMI: Body mass index

## Results

**Figure 1:**
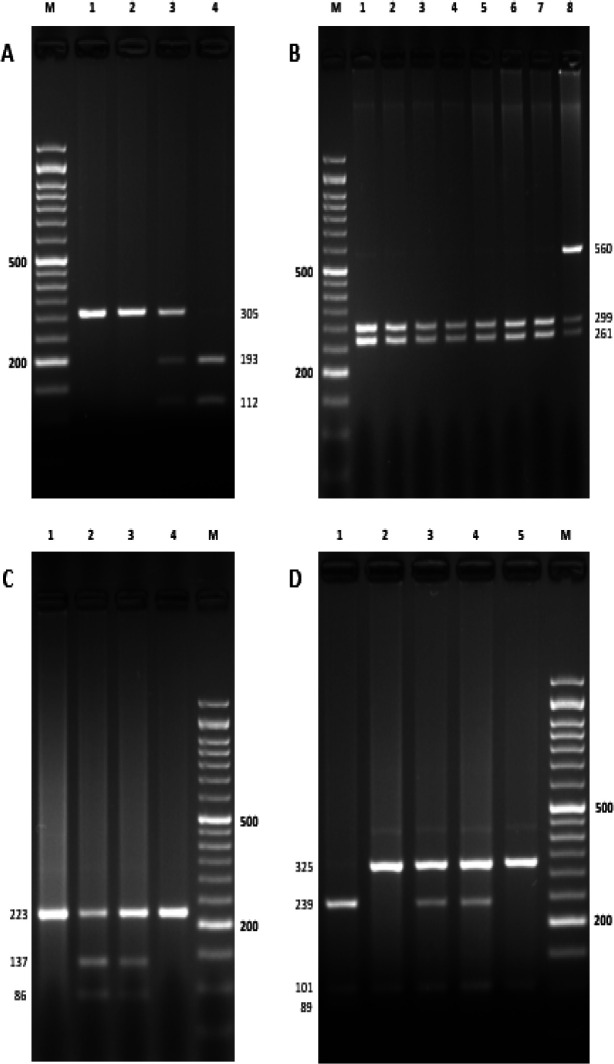
A-D. The digestion patterns of IL-22RA1 (rs4648936 A/G), IL-22 (rs1179251 C/G), IL-22RA1 (rs10794665 A/G), and IL-22 (rs1179246 A/C) single nucleotide polymorphisms. (a) The electrophoresis result of PCR products by BsmAI restriction enzyme on 2% agarose gel at IL-22R rs4648936 A/G SNP; M DNA size marker 50 bp, 1 and 2 genotype GG, 3 genotype AG and 4 genotype AA. (b) The digestion result of PCR products by AlwNI restriction enzyme on 3% agarose gel at IL-22 rs1179251 C/G SNP; M DNA size marker 50 bp, 1-7 genotype CC and 8 genotype CG. (c) The digestion result of PCR products by AluI restriction enzyme on 3% agarose gel at IL-22RA1 rs10794665 A/G SNP, 1 and 4 genotype GG, 2 and 3 genotype GA and M DNA size marker 50 bp. (d) The digestion pattern of PCR products by AluI restriction enzyme on 2% agarose gel at IL-22 rs1179246 A/C SNP; 1 genotype AA, 2 and 5 genotype CC, 3 and 4 genotype AC, M DNA size marker 50 bp

The demographic and clinical features were compared between CRC patients and healthy controls, and the results are presented in Table 2. Among the available clinical data, mean age of CRC cases was significantly older than controls (*p*<0.001) and the median BMI of them was practically identical between cases and controls (*p*=0.112). Additionally, no detectable differences were found between the cases and control subjects according to their gender, BMI, or smoking history. The restriction enzymes digestion results of PCR products at four different single nucleotide polymorphism positions are demonstrated in Figure 1. 

**Figure 2:**
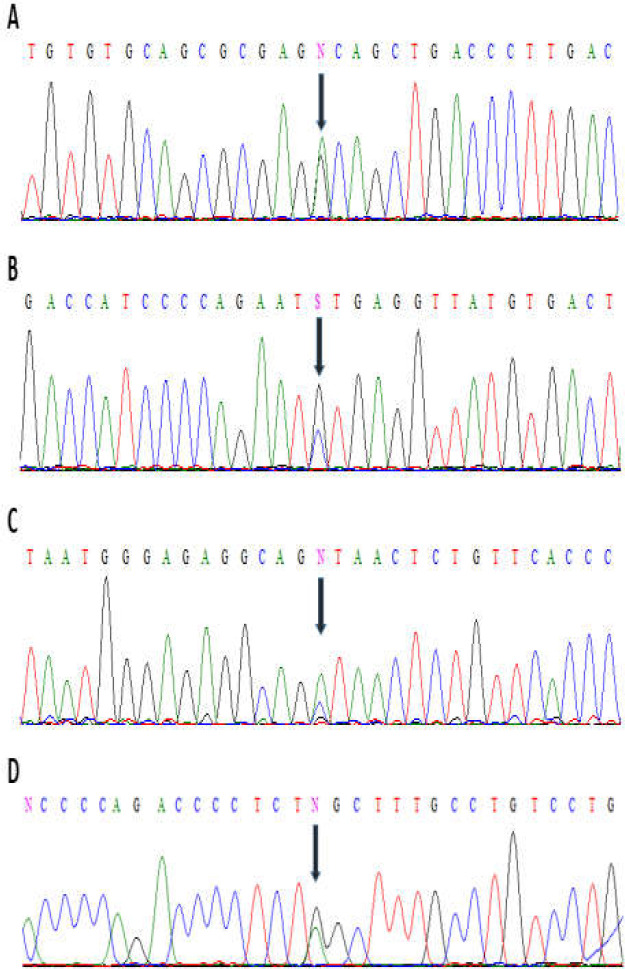
A-D. Results of direct sequencing. Black spears show the DNA sequence of heterozygous A) AG for IL-22RA1 rs4648936 SNP, B) CG for IL-22 rs1179251 SNP, C) AC for IL-22 rs1179246 SNP and D) AG for IL-22RA1 rs10794665 SNP

Final confirmation of genotyping by RFLP tests were performed by rechecking 5% of all samples employing the Sanger sequencing technique. The results of direct sequencing for heterozygous genotypes are shown in Figure 2.

The genotype and the allele distributions for IL-22 gene (rs1179251 and rs1179246) and IL-22Rα gene (rs4648936 and rs10794665) are shown in Table 3. All polymorphic markers studied followed the Hardy-Weinberg equilibrium. None of the genotypes and allele frequencies of 4 SNPs showed any significant association with CRC risk. Adjusting for covariates like age, BMI, and gender did not change the results.

## Discussion

In recent years, several studies have demonstrated that excessive IL-22 was present in human colon cancer (20-22). Additionally it has been reported to contribute to stimulating colonic epithelial cell lines to produce regulatory molecules (IL-10, STAT3), anti-bacterial peptides (β-defensin, calgranulins), mucin (MUC1, -3, -10, and -13), and overexpression of REG Iα (22-27).

The biological effects of IL-22 are related to the expression of IL-22Rα, a receptor expressed on nonimmune cells such as stromal, epithelial cells, and hepatocytes (28). 

The resultant of IL-22 and IL-22Rα complex is able to stimulate transcription of the STAT3 pathway which has been associated with inflammation, proliferation, and protection from apoptosis through phosphorylation of JAK/STAT and MAPK signaling pathways (29-31). The elevated serum level of IL-22 protein or mRNA transcripts were shown in Crohn’s disease, interstitial lung diseases, and rheumatoid arthritis that are correlated with disease severity (32-34). IL-22 also induces acute-phase reactants in hepatocytes and likely has a protective role in liver injury through STAT3 activation (35). Marehalli et al. showed elevated levels of IL-22Rα expression in epithelial cells of the colon cancer cell line (22). Moreover, they found a functional role for IL-22 in intestinal inflammation. IL-22 has also been considered an inflammatory driver in IBD and CRC based upon both clinical evidence and mouse model data (36, 37).

The association of single nucleotide polymorphisms with different hepato-gastrointestinal diseases including colorectal cancer (38-41), gastric cancer (42-45), pancreatic cancer (46-48), hepatitis B virus chronic infection (49-51), hepatitis C virus infection (45, 52-56), and inflammatory bowel disease (57, 58) have been studied for a long time. 

Gao et al. evaluated the links between eight IL22 SNPs (rs1026788, rs2227472, rs2227491, rs2227485, rs1179249, rs2046068, rs2227473, and rs7314777) and the development of chronic HBV cirrhosis in a Chinese Han population. They found differences in genotype and allele distributions of SNPs rs1179249 and rs2227472 between liver cirrhosis patients and chronic patients. In addition, the G alleles of SNP rs2227491 and rs1026788 were observed more frequently in liver cirrhosis patients than in the chronic hepatitis B group (59). 

Furthermore, Wang et al. analyzed the probable links between IL22 gene polymorphisms (rs1179251, rs2227485, rs2227511, and rs2227473) and possible risk of different cancers including liver, lung, and gastric cancers. No association between these SNPs and cancer development can be found (60). 

In the present study, the association of four SNPs in IL-22 and IL-22Rα genes and CRC susceptibility was assessed. For IL-22, two SNPs in intron 4 and the 3’-UTR were studied, and for IL-22Rα two SNPs in 3’near gene and intron 6 were studied. The results showed no significant association between polymorphisms of IL-22 rs1179251 and rs1179246 or between IL-22Rα rs4648936 and rs10794665 before or after adjustment for covariates (gender, BMI, and age) and disease risk. Only a few studies have assessed the association between IL-22 gene polymorphism and the risk of CRC. However, in hepatitis C virus infection (HCV), Branwen et al. showed that the carriage of C allele for IL-22 rs1179251 appeared to have an impact on viral clearance and non-response to treatment (61). In contrast to the current outcomes, Thompson et al. demonstrated an association between rs1179251 in IL-22 with the risk of colon cancer (19). Inappropriate sample size or genotyping error may explain the discrepancy in the results. Therefore, this study should be replicated with a larger sample size to clarify these results. 

To the best of the authors’ knowledge, this is the first investigation to have evaluated IL-22Rα gene variations and its potential role in CRC susceptibility. However, Endam et al. observed that rs4648936 in IL-22Rα is associated with severe chronic rhinosinusitis (62). Obviously, further studies in this area of research are necessary.

There are clear limitations in the current study that should be noted. First is the lack of complete clinical characteristics of cases and control subjects, which may influence the availability of data on covariates. Thus, the current results should be interpreted with prudence. Another limitation is relatively small sample size that prevents representing strong conclusions. Finally, the existence of other polymorphisms outside the studied region that may influence the function of the polymorphisms should not be excluded. Therefore, it may be misleading not to consider other interactive variations.

The findings presented here did not confirm any support for a putative role of genetic variants in IL-22 and IL-22Rα in relation to CRC risk. These results should be replicated in other investigations with appropriate numbers of subjects to further evaluate the potential association between these polymorphisms and CRC susceptibility.

## Conflict of interests

The authors declare that they have no conflict of interest.
